# Cortical network modulations associated with prolonged training of the multiple object-tracking task

**DOI:** 10.1162/imag_a_00577

**Published:** 2025-05-12

**Authors:** Anna-Maria Felßberg, Nadine Schönemann, Jens-Max Hopf, Mircea Ariel Schoenfeld, Christian Merkel

**Affiliations:** Department of Neurology, Otto-von-Guericke University Magdeburg, Magdeburg, Germany; Leibniz-Institute of Neurobiology, Magdeburg, Germany; Schmieder Kliniken, Heidelberg, Germany

**Keywords:** multiple-object tracking, object-based attention, multivariate pattern analysis

## Abstract

A fundamental mechanism enabling object permanence for the visual system constitutes visual tracking. During the interaction with a dynamic visual environment we are able to continuously track a multitude of objects simultaneously. Early work suggests that this mechanism is subject to improvement under task-specific behavioral training, though exhibiting a limited transferability to other cognitive tasks. Interestingly enough, specific groups of subjects, regularly involved with demanding visual tasks, may possess expertise in the tracking-task, hinting toward long-term perceptual learning processes playing a role in modulating functional networks involved in visual tracking. In order to identify functional networks being susceptible to cortical flexibility during prolonged task-specific performance, thirty-three subjects executed a multiple-object-tracking task over the course of five successive sessions over 2 weeks. During the first and last session, domain-specific location- and object-based representational functional response patterns toward the relevant, attended target objects at the end of the tracking phase were recorded using a 3T Scanner. Differential modulations were observed within two separate functional networks identified previously being involved in encoding the location-based and object-based aspects of the tracking task. During training, parametric, location-based information processing consolidates preferentially within visual cortical areas over time and shifts from an object-based, non-parametric mechanism within frontal control networks.

## 1 Introduction

We continuously engage with a complex visual dynamic environment, which requires the maintenance of a multitude of simultaneously relevant objects, in order to select goal-directed responses. The effective perception and processing of visual input is flexible and continuously shaped by the intraindividual history of visual experiences (Daw et al., 2006;Jansma et al., 2001;Sutton & Barto, 2018).

To operationalize the dynamic aspects of a crowded visual environment, the multiple-object tracking (MOT) paradigm has been introduced byPylyshyn and Storm (1988), who impressively demonstrated the capabilities of the human visual system to maintain location-information of multiple identical moving visual objects across longer periods of time. Surprisingly, most subjects are able to visually track around four fast moving target objects among a number of distractor objects with very high accuracy (Alvarez & Franconeri, 2007;Pylyshyn, 1989,^2004^;Scholl et al., 2001). This feature of the visual system is difficult to reconcile with classical approaches of spatial attention (Treisman & Gelade, 1980). Resource allocation by spatial attention is thought to be limited in that the locus of attention is spatially indivisible (Eriksen & St. James, 1986; Özkan & Störmer,^2024^;Posner, 1980) and shifts among targets are constrained by an upper temporal limit (Carlson et al., 2006;Hogendoorn et al., 2007;Holcombe & Chen, 2013;Pylyshyn, 1989). Such limitations are thought to parametrically modulate the cognitive strain on a central spatial resource (location-based approach) depending on the spatial load of the task defined by the number of relevant targets, item-speed, or inter-item distances (Alvarez & Franconeri, 2007;Chen et al., 2013;Franconeri et al., 2010;Shim et al., 2008). Moreover, multiple object tracking can be facilitated by object-based mechanisms operating on an abstract configuration formed by the entire set of target objects (Merkel et al., 2014,^2017^,^2024^;Yantis, 1992). Those two, location-based and object-based tracking processes elicit different electrophysiological signatures (Merkel et al., 2014,^2022^) and are implemented within partly distinct functional networks (Merkel et al., 2015).

Over longer periods of performing the object-tracking task, the performance improves in a task-specific manner (Faubert & Sidebottom, 2012;Legault et al., 2013), unexpectedly showing only little transfer to other non-spatial tasks (Oksama & Hyönä, 2004;Thompson et al., 2013).

Interestingly, some expert participants such as air-traffic controllers (Allen et al., 2004), first-person video game players (Green & Bavelier, 2006), and team sport players (Zhang et al., 2009) exhibit very high accuracies even during their first performance of the task. This suggests that individual long-term perceptual experiences can facilitate processes, which are beneficial in successfully executing the object-tracking task.

With the current work, we sought to identify the neural networks that are displaying cortical flexibility elicited by practicing the multiple object-tracking task. For this purpose, we examined changes in the parametric and non-parametric neural encoding of visual probes at the end of the tracking phase that differently match the number of previously tracked objects prior and after practicing the object-tracking task. We expect neural functional patterns to exhibit a parametric or non-parametric variation based on a preferential location- or object-based encoding of the representation of the relevant target objects. Domain-specific changes of those patterns would indicate how training affects tracking on a functional network level.

## 2 Methods

### 2.1 Subjects

Forty-six participants were initially recruited for the current study approved by the ethics board of the Otto-von-Guericke University (no. 141/20). All subjects gave written informed consent and were paid for their participation. Tested participants reported normal or corrected-to-normal vision and were right-handed with the exception of one subject. Nine subjects were subsequently excluded due to excessive motion artifacts during the MR-scanning. An additional three subjects had to be removed due to technical difficulties or noncompliance during the task respectively. The data of one subject who did not conclude the last session of the current multi-session design in the set time frame (see ‘Procedure’) were also excluded. The remaining 33 analyzed participants included 16 females and had a mean age of 28.64 (sd = 4.58).

G*Power a-priori calculations for sample size suggest 34 subjects to be sufficient to detect medium effect sizes with a significance level of 0.05 and a power level of 0.8., which were detected in a previous study investigating the processing of two distinct domains (spatial and object-based) during object-tracking (Merkel et al., 2015). Sample size estimations were performed for two-factorial repeated-measures ANOVAs covering the time-dimension for the training and the functional dimension for processing spatial- and object-based representations.

### 2.2 Procedure and task

**Fig. 1.**
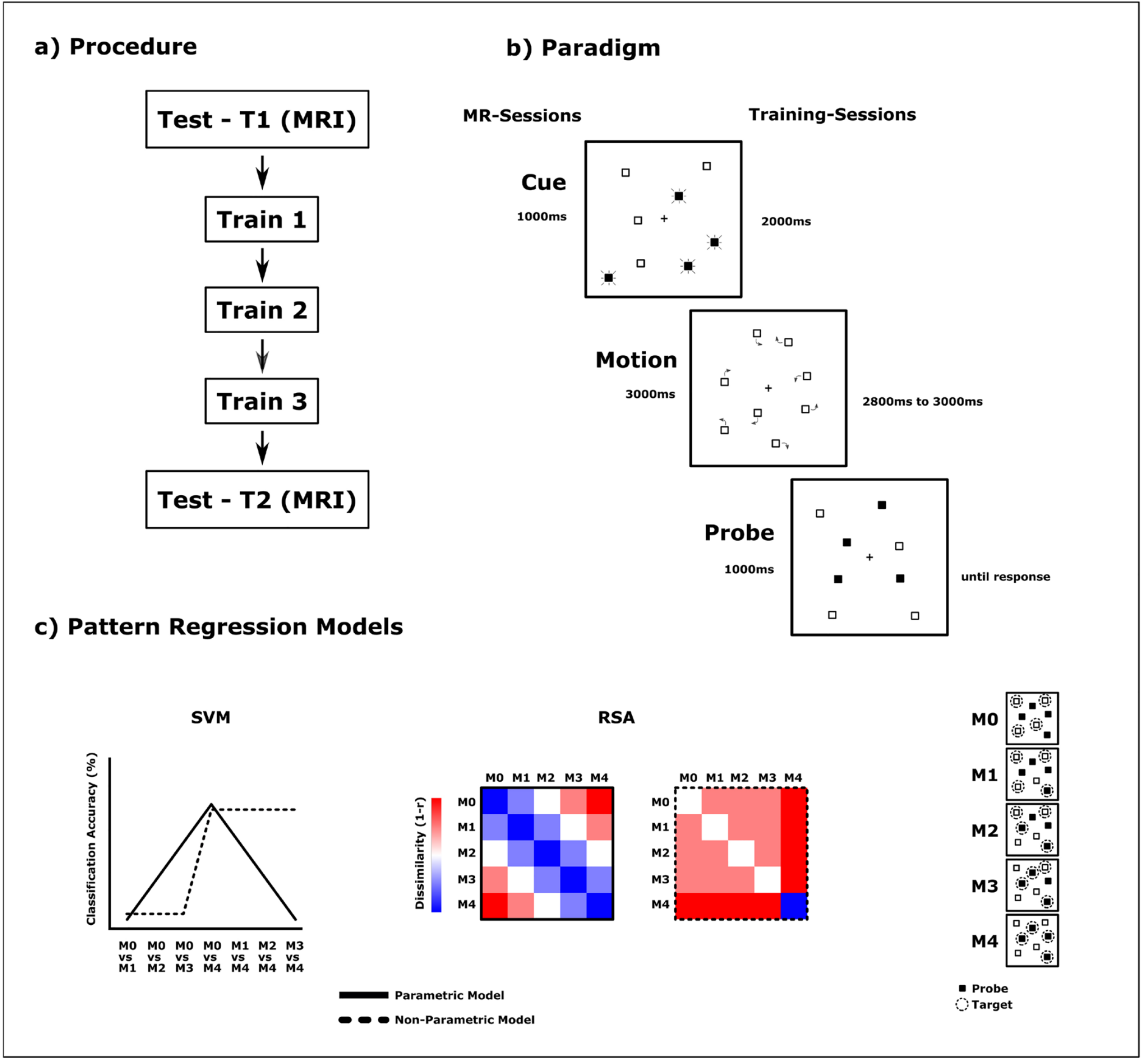
Task and Study Design. (a) In a longitudinal design, subjects performed the tracking task on five consecutive sessions, which were spaced in between 1 and 4 days apart. The first and last sessions were used as testing sessions (T1/T2) to measure performance in the tracking task, while recording the functional BOLD. The intermittent three sessions were used to train subjects in the object-tracking task. (b) For the actual task, subjects were required to simultaneously keep track of four out of eight visually identical items, while all of them moved randomly through the visual field for up to 3 seconds. During a cueing-phase, the four relevant items for the present trial were indicated. During the subsequent motion phase, all items moved around randomly after which again four items were highlighted and subjects had to respond, whether all of the four highlighted (probed) items were previously cued (target) (M4) or not (M0/M1/M2/M3). (c) The functional patterns elicited by the probes were analyzed using multivariate methods. Classification and dissimilarity results were regressed against a parametric and non-parametric model indicating location-based or object-based processing mechanisms, respectively.

All subjects performed in five separate sessions individually spaced over the course of up to 2 weeks (Two consecutive sessions were conducted at least 1 day and not more than 4 days apart) (Fig. 1a). The first (T1) and fifth session (T2) were performed in a 3T Siemens Prisma scanner in order to assess functional activity changes as a result of training. The three intermittent sessions between (T1 and T2) were used to train subjects on the tracking task and were conducted outside the scanner on a separate computer.

The general task for all subjects during the training and scanning sessions was similar and followed the multiple-object tracking paradigm described previously (Merkel et al., 2014,^2015^,^2020^) (Fig. 1b): Stimulus material was constructed and presented using the Psychtoolbox 3 (Brainard, 1997) on MATLAB 2021b (Mathworks, Natick, MA, USA). Stimuli were shown in white on a black background. A white fixation cross (0.5° x 0.5°) was presented in the center of the screen, and subjects were required to maintain fixation throughout the experiment. At the start of each trial, eight initially visual identical hollow squares (0.3° x 0.3°) with white outline appeared on the screen at random locations with four of them being highlighted by filling them white in order to identify them as relevant targets for the current trial. Once all eight items were visually identical again, they moved around the screen on unpredictable but smooth trajectories that were calculated offline beforehand (seeMerkel, 2020). During the movement, items never occluded each other and kept a minimum inter-item-distance of 1.05° of visual angle. Item movements were restrained within an aperture of 17.98° x 17.98° centered on fixation. Trajectories were calculated in a way that items never left this predefined aperture but also did not bounce of its illusory inner border. Movement lasted for up to 3 seconds with a speed of 4.3°/seconds, after which all items stopped with four of them being highlighted again, constituting the probe. Subjects had to keep track of all four relevant objects throughout the motion-period as accurately as possible, while maintaining fixation. Once the motion ceased and the probe (highlighting four items) appeared, the subjects’ task was to respond, whether the set of all four probe items matched of all four relevant target items (full-match) or not in a 2-alternative forced choice task. Across five possible conditions, the probe could consist of only distractors (M0), or one to four targets (M1/M2/M3/M4). The response was given using the right index and middle finger with the response-assignments for full-match (M4)/no-full-match (M0/M1/M2/M3) counterbalanced across subjects. Participants were required to respond toward the probe display as fast as possible.

Prior to the first session performed in the scanner, subjects executed a short training in order to become familiar with the task. For the scanner-sessions, subjects performed overall 470 trials over 8 runs. The stimulus material was projected into the bore by a mirror and a back-projection screen. The cue was presented for 1000 ms, and the tracking phase lasted for exactly 3 seconds. The probe appeared for 1 second on the screen. Stimulus timings for the training sessions were slightly different. The cue was presented for 2 seconds, and the motion phase had a duration of 2800 to 3000 ms. The probe was present on the screen, until a response was selected. Subjects performed 300 trials within each training session. The offline-calculated trajectories were different for scanning sessions and training sessions.

### 2.3 Image acquisition

Scanner sessions were conducted using a 3T MRT scanner (Siemens Prisma, Erlangen, Germany). The recording of the first session started with the acquisition of a T1-weighted MP2RAGE wholehead image with 0.8 mm isotropic resolution (TR = 5500 ms, TE = 3.28 ms, FoV =256 mm). At the beginning of the second scanner session, a structural MPRAGE image was recorded (1.0 x 1.0 x 1.0 mm, TR = 2500 ms, TE = 2.82 ms, FoV = 256 mm).

Using a T2 echo-planar pulse sequence with 36 slices (3.0 x 3.0 x 3.0 mm, FoV = 240, TR = 2000 ms, TE = 30 ms, FA = 90°), overall 1920 functional scans (240 per run) were acquired during the task in each scanner session.

### 2.4 MRI-preprocessing

The cortical surface of each subject was reconstructed based on the anatomical MPRAGE scan using FreeSurfer (Dale et al., 1999;Fischl et al., 1999). Surface normalization was performed in order to transform individual functional searchlight and regression maps (see classification analysis) into overlay maps and projection onto the fsaverage standardized FreeSurfer surface.

**Fig. 2.**
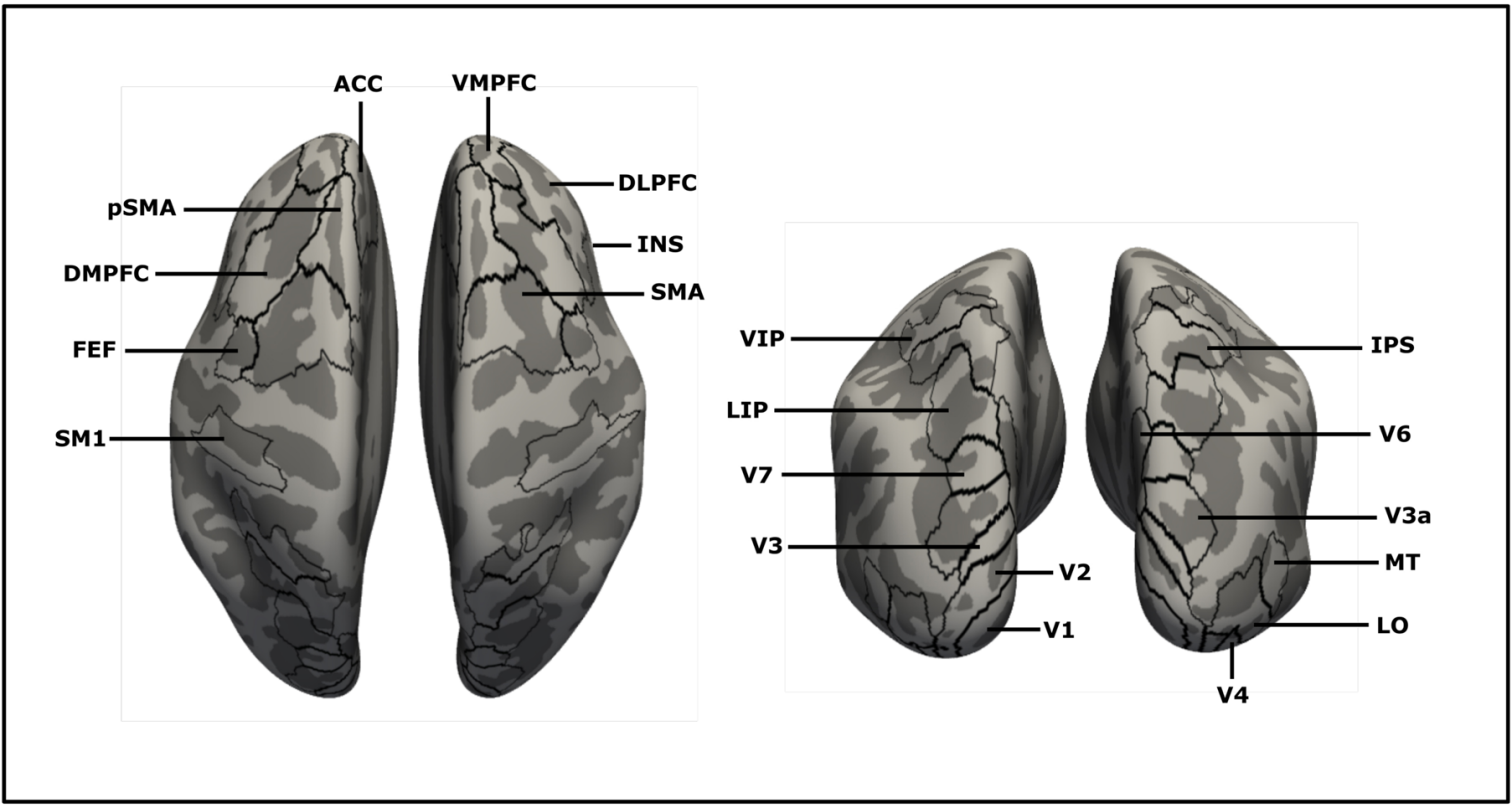
ROI-definitions across the normalized structural cortical surface. Classification data were analyzed within predefined regions of interest (ROI). Those ROIs were extracted from a combination of cortical parcellations proposed by Glasser and Sereno (Glasser et al., 2016;Sereno et al., 2022) and covered frontal, parietal, and occipital regions.

A combination of the HCP-MMP1 atlas (Glasser et al., 2016) and the CsurfMaps1 (Sereno et al., 2022) atlas was used to define specific functional cortical regions-of-interest (ROI) within the occipital, parietal, and frontal lobes inside the fsaverage-surface space. Second-level analyses were conducted within 21 selected ROIs within the frontal cortex (FEF, SMA, pSMA, DLPFC, ACC, INS, DMPFC, VMPFC), parietal cortex (LIP, IPS, VIP), extrastriate occipital cortex (V3a, LO, V4, V6, V7, MT), early visual cortex (V1, V2, V3), and the primary sensory-motor hand area (SM1) (Fig. 2).

### 2.5 fMRI-preprocessing

Preprocessing of the functional data of each scanning session was performed using SPM12 (Welcome Department of Cognitive Neurology, University College London, UK) and included slice time correction of each individual scan by temporally aligning each of the 36 slices to the first slice of that scan. This was followed by a motion correction spatially realigning each runs scans to the mean of that run and later to the mean of all runs. Additionally, the functional scans were coregistered across sessions with the first anatomical scan.

### 2.6 Parametric and non-parametric response patterns

The match conditions in the current study differ in their quantitative overlap between the probed items and the target items as higher match conditions would contain more attended items. We propose a spatial attentional mechanism matching the target and probe items individually at the time the probe appears. Accordingly, within a higher-dimensional representational space, the five match conditions would thus be linearly (parametrically) aligned. On the other hand, considering evidence for an object-based attentional mechanism operating on the configuration of the entire target-set, a different placement of the match conditions within that same representational space can be formulated, with the full-match (M4) condition being highly distinct from any of the other match condition. Only probe displays containing exclusively targets would be represented as an abstract object, compared to any other probe condition.

Multivariate analysis approaches were applied to test for the degree of fit between observed functional multi-voxel response patterns toward the different match conditions within different areas and those two proposed parametric or non-parametric representational model patterns. The estimated model parameters for the parametric and non-parametric patterns are, thus, a quantification of a spatial versus object-based representation of tracked items at the moment the probe is presented.

#### 2.6.1 SVM-analysis

First, we conducted pairwise classification analyses using a support-vector-machine (SVM) algorithm implemented in PyMVPA (Hanke et al., 2009). The classifications were performed on the functional raw data following each match display. Note, that the functional response toward the visual probe allows for an inference about the prior target set which varies in congruity with the following probe. The second and third scan following each probe were extracted and averaged using FSL-scripts (Smith et al., 2004), yielding 470 functional volumes containing neural responses for all trials toward each of the five match conditions within each of the two sessions. At each voxel across the functional volumes, the signal was subsequently de-trended and z-scored. While most of the sluggish BOLD signal at that latency would contain responses related to the cueing and tracking phase itself, it is critical to acknowledge that trials do not differ during the cueing and motion phase of the trial as always four out of eight items have to be tracked. The match condition itself is only defined by the congruity of the probed items with the relevant target items at the end of each trial.

Pairwise classifications were performed in a whole-brain fashion using the searchlight-approach with a radius of 5 mm (Kriegeskorte et al., 2006) for each subject at each scanning session (T1/T2). Classification estimates were cross-validated using the neural responses of trials of each run as a validation dataset while training the SVM on the data of all other seven runs respectively. Seven pairwise classifications were performed, yielding seven searchlight maps for each subject and session (M0 vs. M1 / M0 vs. M2 / M0 vs. M3 / M0 vs. M4 / M1 vs. M4 / M2 vs. M4 / M3 vs. M4).

The results of those seven pairwise classifications are best suited to compare with the parametric and non-parametric representational models (Fig. 1c, left). Classification accuracies mirror the distance between the full mismatch (M0) and any other condition and the full match (M4) and any other condition in the representational space. In this model, parametric encoding would in this case cause a linear variation of pairwise classification accuracies with a maximum of discriminability between M4 and M0. In contrast, a non-parametric representation encoded within the functional pattern would result in poor discrimination between individual mismatch conditions, while all mismatch conditions would exhibit an equally high discriminability toward the full-match condition. The observed classification performances were compared with the parametric and non-parametric model using a regression of the seven discrimination values as dependent variable [M0 vs. M1 / M0 vs. M2 / M0 vs. M3 / M0 vs. M4 / M1 vs. M4 / M2 vs. M4 / M3 vs. M4] and of the two modeled (z-scored) regressors as independent variables: parametric [1 2 3 4 3 2 1], non-parametric [1 1 1 2 2 2 2]. The regression was conducted on the seven searchlight maps, resulting in two standardized beta maps quantifying the degree of parametric and non-parametric representation of probed objects across the cortex for each subject at each scanning session.

#### 2.6.2 RSA-analysis

As a second independent multivariate approach, we performed a correlation analysis of the functional responses between any two match conditions as a representational similarity analysis (RSA). This yielded representational dissimilarity matrices (RDM) per subject and session, which were then regressed against two model RDMs capturing parametric and non-parametric representational relationships of the match conditions.

First, match condition responses were not entered as raw signals as in the SVM analysis but were first modeled as beta maps using univariate general linear modeling (Kriegeskorte, 2008). Each condition of each run was modeled as a separate regressor. Head motion parameters and linear drifts were included as covariates to remove noise from the model. RDMs were calculated using the correlation of condition estimates between different runs (Mumford et al., 2014). A searchlight-approach yielded RDMs for each voxel. Cells of each RDM were collapsed across the same pairs of match conditions (independent of run), resulting in 5 x 5 RDMs. Entries above and including the diagonal were used as the dependent variable for a regression with the two modeled parametric and non-parametric variables across the cortex and within the predefined anatomical ROIs. At each cell of the parametric RDM model, the similarity value equals the ordinal distance between the corresponding two match conditions. The non-parametric model RDM contains maximal dissimilarity values for any full-match comparisons and weak dissimilarities for any two partly mismatch conditions (Fig. 1c, right).

#### 2.6.3 GLM-analysis

As a final test, scrutinizing the multivariate encoding of the match and mismatch information between probe and target within the functional response patters, we performed a univariate RSA between the match conditions. We used the RSA-pipeline described above with the only difference that similarity between conditions was not quantified as correlation between voxel-patterns but by the simple difference of the mean beta values across the pattern. This analysis is equivalent to a standard GLM as it quantifies absolute activation difference between conditions.

### 2.7 Analysis

Behavioral responses toward the different conditions were analyzed for the first-scanning session (T1) and second scanning session (T2) using one-factorial (‘match’) and two-factorial (‘match’ x ‘time’) repeated-measures ANOVAs in order to carve out specific behavioral patterns toward the conditions and the potential training effect over time.

Results for the model regressions were plotted on the tksurfer surface. Beta values for the parametric- and non-parametric-regressors across subjects for the first (T1) and second (T2) session were tested for significance at each surface location using univariate t-tests to illustrate parametric- and non-parametric–related distributed functional networks.

Multi-factorial rANOVAs including factors of ‘regressor’, ‘time’, ‘hemisphere’, and ‘roi’ were conducted to identify functional ROIs showing specific parametric and non-parametric related functional patterns as well as a differential susceptibility of those functional patterns to behavioral training within certain ROIs. Multivariate tests were Greenhouse-Geisser-corrected.

Additionally, a behavioral combination score was calculated at each time point and for each subject across conditions taking error-rates and reaction times into account (balanced integration score—BIS) (Liesefeld & Janczyk, 2019;Liesefeld et al., 2015). The general performance as well as its change over time induced by training was correlated with the beta-values for the parametric and non-parametric regressors at each time point as well as with the regressors’ changes, using the Pearson coefficient. Thus functional correlates for the initial MOT-performance as well as performance increase were analyzed.

## 3 Results

**Fig. 3.**
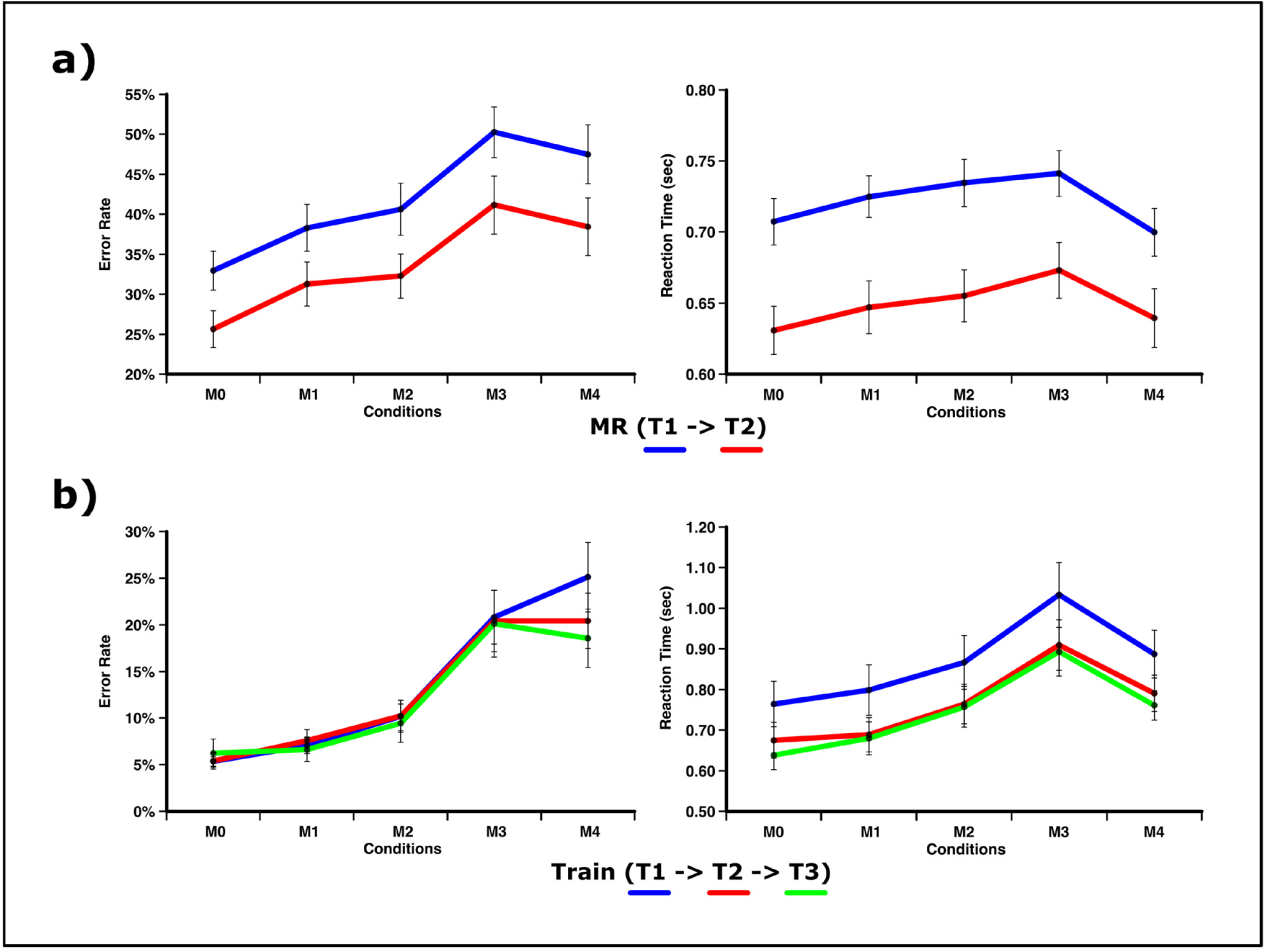
Behavioral performance for the testing and training sessions. (a) At each testing session, performance decreased with increasing match between probe and target except for the full-match condition. For both, error rate and reaction time, subjects showed a marked improvement from the first to second testing session across all match conditions. (b) Training effects during the training session emerge mostly as an improvement in error rates for the full-match and a general decrease in reaction times across all match conditions.

During the first round of executing the multiple object-tracking task (T1), subjects exhibited the previously observed pattern of decreasing performance with increase in target-probe mismatch across conditions (Fig. 3a). At the same time, performance toward the full match condition deviated from this pure monotonic trend showing significant enhancement. A one-factorial repeated-measures ANOVA testing for differences in error-rates at T1 revealed a significant effect across all five probe-match conditions (F(4,128) = 10.056, p < 0.001, η^2^= 0.239). At the same time, error-rates in the full match M4-condition did not differ from the M3-condition (t(32) = -0.571, p = 0.572, η^2^= 0.010). Reaction times likewise showed a main effect for probe-match conditions (F(4,128) = 9.963, p < 0.001, η^2^= 0.237). Subjects responded faster toward the full-match condition compared to the M1-condition (t(32) = -2.784, p = 0.009, η^2^= 0.195) but not the M0-condition (t(32) = -0.767, p = 0.449, η^2^= 0.018).

In order to examine the behavioral effect of the training, changes in performance across the two MR-sessions (T1->T2) were examined using a two-factorial repeated-measures ANOVA, including the factors match-condition (M0/M1/M2/M3/M4) and session (T1/T2). As during the first MR-session, error-rates and reaction times differed between match conditions (ER: F(4,128) = 12.889, p < 0.001, η^2^= 0.287; RT: F(4,128) = 11.050, p < 0.001, η^2^= 0.257). Additionally, both performance measures exhibited a strong improvement over time (ER: F(1,32) = 13.104, p < 0.001, η^2^= 0.291; RT: F(1,32) = 48.895, p < 0.001, η^2^= 0.607), indicating a clear training effect. Importantly, this behavioral training effect was present across all conditions (ER: F(4,128) = 0.202, p < 0.817, η^2^= 0.006; RT: F(4,128) = 1.875, p < 0.137, η^2^= 0.055) and thus seems to be unspecific with respect to object- or location-based selection processes at the time of probe presentation.

Behavioral data for the training sessions outside the scanner were acquired within a partly different setting. Therefore, results from both experiments cannot be directly compared, since training effects of object tracking do not fully transfer across different designs (Strong & Alvarez, 2017). However, the training effect itself can still be observed within the behavioral training (Fig. 3b). The reaction times decrease overall during the three training sessions (F(2,60) = 8.447, p = 0.003, η^2^= 0.220) with the subjects getting faster early on (T1->T2: F(4,120) = 11.628, p = 0.002, η^2^= 0.267, T2->T3: F(4,120) = 0.223, p = 0.640, η^2^= 0.007). The pattern of the errors, however, mostly improves for the full-match condition during the training sessions in a fairly steady rate (T1->T2: time t(32) = 1.975, p = 0.057, η^2^= 0.109, T2->T3: t(32) = 2.029, p = 0.051, η^2^= 0.121).

A main objective of the current study was to examine the cortical distribution of functional networks engaged in either representing individual item positions or abstract object configurations after probing the tracking of multiple objects and monitoring potential changes within those networks over time.

Distances between single conditions in representational space were quantified using different analysis techniques, including prediction accuracy (SVM), correlation (RSA), and mean activation difference (GLM). The patterns of condition distances were regressed against two models. One served to gauge a parametric variation across match-conditions which indicates single-item processing (parametric model) and a second model tested the full-match condition associated with abstract object representation (non-parametric model).

**Fig. 4.**
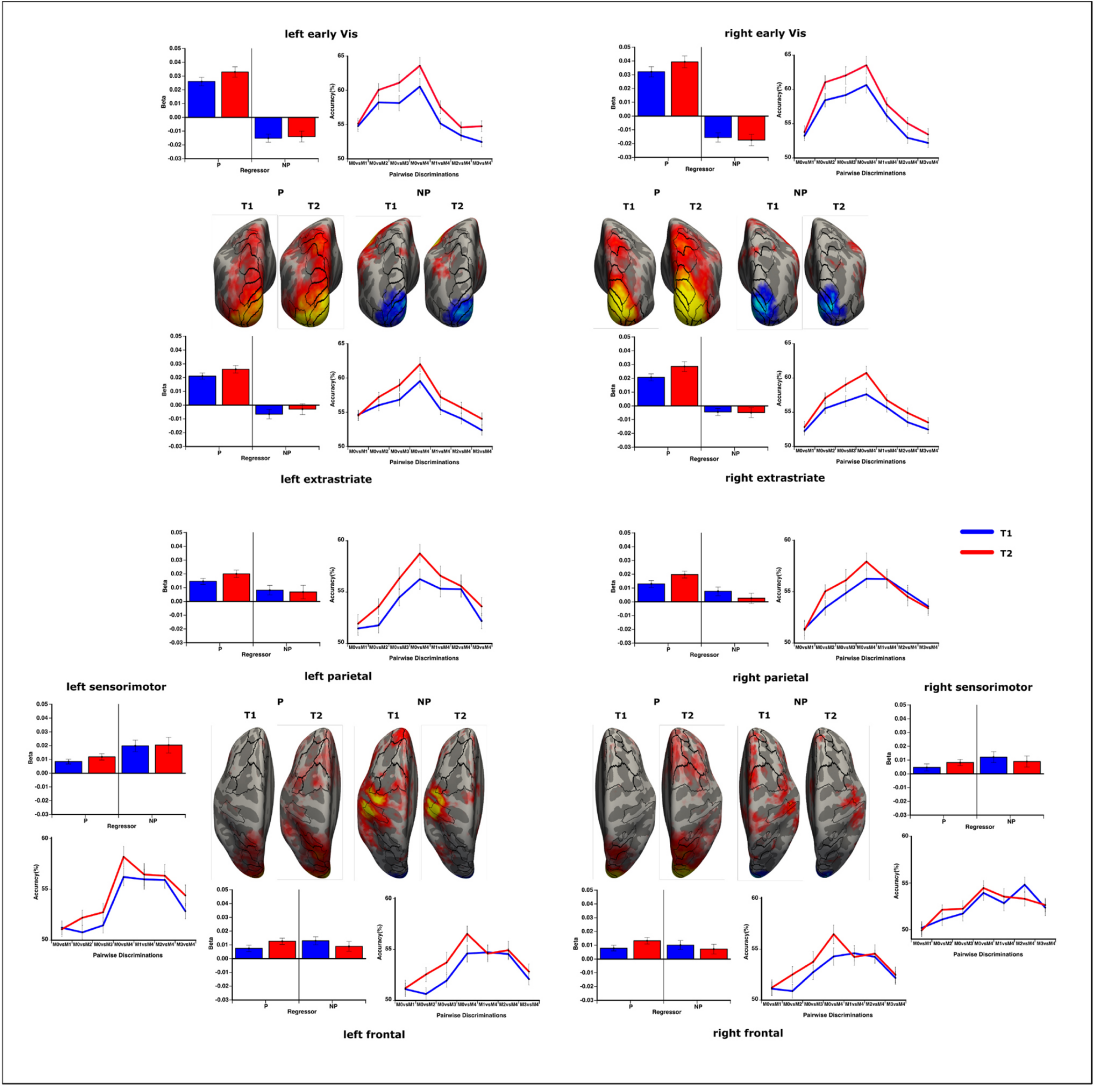
Classification results for the SVM analysis and following regression. Classification accuracies between pairwise comparisons and regression of these classification patterns against the parametric and non-parametric model for each region and scanning session are shown. Additionally, the regression model was performed for each vertex across the cortex and beta-values for the inflated normalized surface are displayed. A generally better fit for the parametric model of the classification pattern can be observed within posterior regions, whereas the non-parametric fit increases within higher cortical areas. The parametric model fit increases over time and shows a clear right-hemispheric bias. Within frontal areas, a shift from classifying full-match versus partly matching conditions toward a discrimination between all conditions can be seen over time.

For the SVM-approach, we first examined the underlying accuracy distributions of the pairwise classifications within each region across hemispheres and scanning sessions using three-way repeated-measures ANOVAs (classifications x hemi x time). Subsequently, differences in the fit for the two model predictions (parametric/non-parametric) with the classification profiles were analyzed using rANOVAs, including the factors ‘regressor’, ‘hemisphere’, and ‘time’ (Fig. 4). The primary sensorimotor area is an ideal example for this approach, showing large differences in pairwise classification profiles due to separate motor responses being assigned to the full match condition (right index finger) versus the partly matching condition (right middle finger). Accuracies differ between classifications (F(6,192) = 21.508, p < 0.001, η^2^= 0.402) but most importantly across hemispheres (F(1,32) = 16.157, p < 0.001, η^2^= 0.336), with the left motor-cortex showing high accuracies especially for the full-match classifications (F(6,192) = 7.340, p < 0.001, η^2^= 0.187). Regressing the profiles against the model predictions highlight these results, showing a better fit for the left hemisphere (F(1,32) = 25.645, p < 0.001, η^2^= 0.445) and the non-parametric model (F(1,32) = 4.375, p = 0.044, η^2^= 0.120). The beta-values do not change over time in this region (F(1,32) = 0.139, p = 0.712, η^2^= 0.004).

Within the early visual region (V1/V2/V3), a clear variation in classification accuracy can be observed (F(6,192) = 46.156, p < 0.001, η^2^= 0.591) where accuracy increases with the dissimilarity in target-probe congruity between matches. This classification profile differs between left and right hemisphere (F(6,192) = 9.253, p < 0.001, η^2^= 0.224) while overall accuracy across hemispheres remains identical (F(1,32) = 0.003, p = 0.959, η^2^= 0.000). Classification is generally better in the second relative to the first scanning session (F(1,32) = 12.993, p = 0.001, η^2^= 0.289). The parametric model shows highly positive beta values, while the non-parametric model exhibits a significant negative fit (F(1,32) = 73.846, p < 0.001, η^2^= 0.698) with a strong right-hemispheric bias for the linear fit (F(1,32) = 15.175, p < 0.001, η^2^=0.322). Over time, the parametric model fit increases across hemispheres (F(1,32) = 4.847, p = 0.035, η^2^= 0.132). The non-parametric fit does not change over time (F(1,32) = 0.012, p = 0.912, η^2^< 0.001).

The extrastriate visual areas (V3a/V4/V6/LO/MT/V7) exhibit variations in pairwise discriminations (F(6,192) = 30.741, p < 0.001, η^2^= 0.490) with a general increase over time (F(1,32) = 8.923, p = 0.005, η^2^= 0.218) as well. Classification performs better within the left hemisphere (F(1,32) = 6.209, p = 0.018, η^2^= 0.162). The parametric model fits the classification data at T1 (F(1,32) = 66.572, p < 0.001, η^2^= 0.675) and shows enhancement over time (F(1,32) = 5.876, p = 0.021, η^2^= 0.155).

While accuracies vary between pairwise classifications within parietal areas (LIP/IPS/VIP) (F(6,192) = 17.823, p < 0.001, η^2^= 0.358), the increase toward the second scanning session only shows a trend (F(1,32) = 3.370, p = 0.076, η^2^= 0.095) which is slightly enhanced for the left hemisphere (F(1,32) = 3.876, p = 0.058, η^2^= 0.108). The regression model, however, confirms an increase for the parametric model over time (F(1,32) = 5.044, p = 0.032, η^2^= 0.136). At T1, both the parametric (t(32) = 6.293, p < 0.001, η^2^= 0.329) and non-parametric model (t(32) = 2.587, p = 0.014, η^2^= 0.173) show a positive fit with the classification profile.

The frontal cortex (FEF/SMA/pSMA/DLPFC/ACC/INS/DMPFC/VMPFC)) exhibits variations in classification accuracies (F(6,192) = 16.193, p < 0.001, η^2^= 0.336) without a clear overall increase in performance over time (F(1,32) = 2.788, p = 0.105, η^2^= 0.080). While the interaction between pairwise classifications and scanning session is not significant (F(6,192) = 1.261, p = 0.286, η^2^= 0.038), the model fit shows a trend toward an interaction between session and regressor (F(1,32) = 3.059, p = 0.090, η^2^= 0.087) with an enhancement over time (F(1,32) = 3.881, p = 0.058, η^2^= 0.108) for the parametric regressor. As within the parietal regions, both parametric (t(32) = 3.622, p = 0.001, η^2^= 0.291) and non-parametric (t(32) = 3.964, p < 0.001, η^2^= 0.553) models show a significant fit with the accuracy profiles at T1.

Including all ROIs from posterior to anterior in a multifactorial rANOVA, there is a clear pattern. The parametric model fit decreases toward frontal areas (F(20,640) = 27.741, p < 0.001, η^2^= 0.464, linear contrast: F(1,32) = 46.372, p < 0.001, η^2^= 0.592), while the non-parametric model fit enhances toward anterior regions (F(20,640) = 29.084, p < 0.001, η^2^= 0.476, linear contrast: F(1,32) = 63.422, p < 0.001, η^2^= 0.665). The parametric model shows a strong right hemispheric bias within the visual cortex (F(20,640) = 5.196, p < 0.001, η^2^= 0.140), whereas the non-parametric model exhibits a left-hemispheric bias within anterior areas (F(20,640) = 3.025, p = 0.003, η^2^= 0.086) (with the left sensorimotor areas contributing to this effect – excluding M1: F(20,640) = 2.589, p = 0.013, η^2^= 0.075). Only the parametric model shows a clear training effect with an increased fit over time across ROIs (F(1,32) = 8.237, p = 0.007, η^2^= 0.205).

**Fig. 5.**
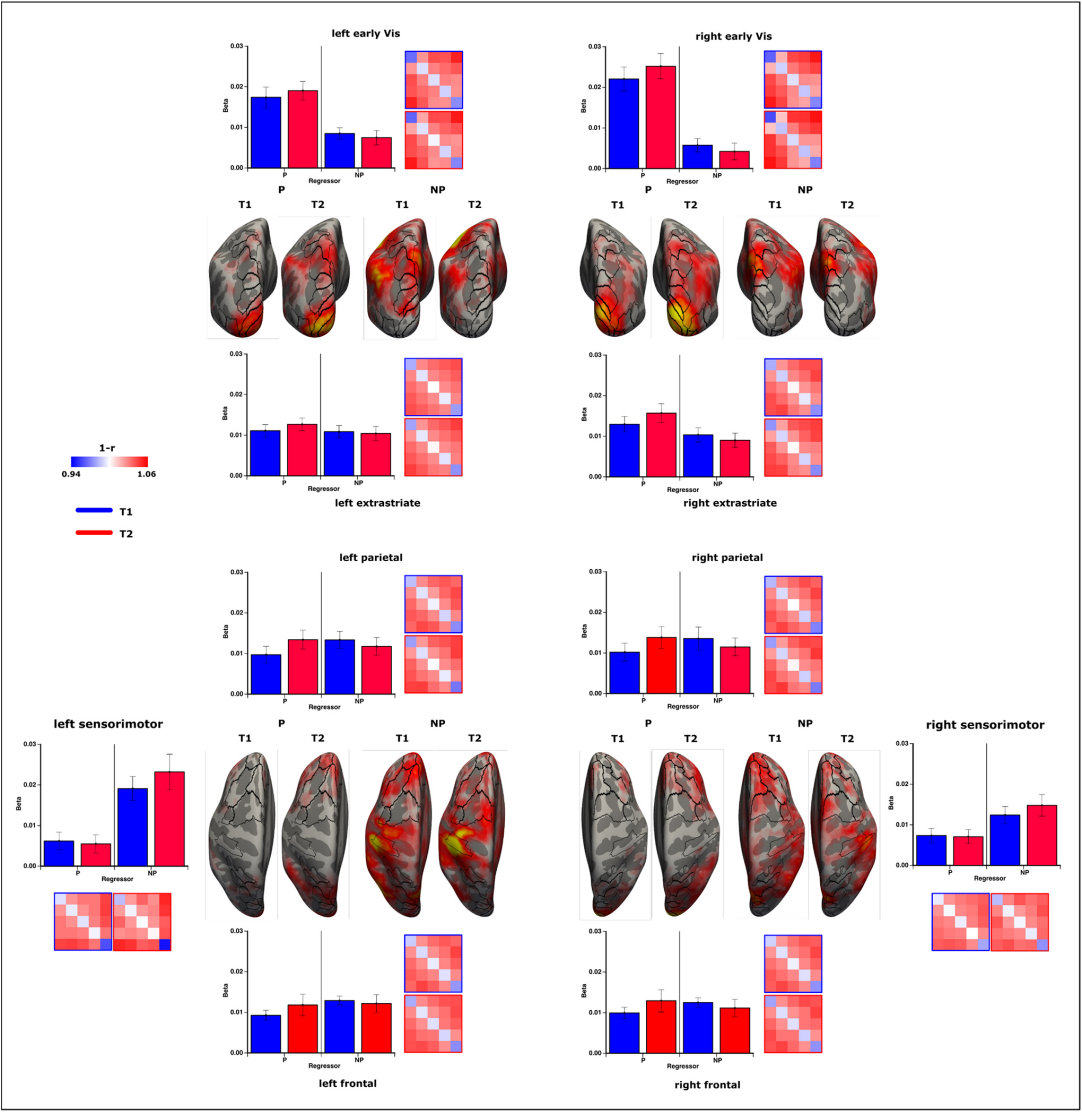
Representational similarity analysis and following regression. Displayed are the representational dissimilarity matrices containing the pairwise distances of the match conditions within higher-order representational space at each cortical region. The fit between those RDMs and the two parametric and non-parametric model RDMs are presented next to them. The RSA results mostly mirror the SVM regressions with a spatial decrease of the parametric model toward frontal areas, while non-parametric representations increase anterior. Over time, the same shift from non-parametric to parametric model fits can be observed.

For the representational similarity analysis the representational dissimilarity matrix was calculated using 1-r between the functional patterns associated with any of the five conditions. The matrices for each ROI were then regressed against the parametric and non-parametric model. Qualitatively, the RSA yields results that are comparable to the SVM (Fig. 5). Across the cortex, the parametric model shows a better fit within the visual cortex (F(20,640) = 5.064, p < 0.001, η^2^= 0.137) and for the right hemisphere (F(1,32) = 14.826, p = 0.001, η^2^= 0.317). The parametric fit does decrease toward the frontal cortex (F(20,640) = 7.883, p < 0.001, η^2^= 0.198). However, the time effect is not significant (F(1,32) = 1.402, p = 0.245, η^2^= 0.042). The non-parametric regressor exhibits a strong left hemispheric bias (F(1,32) = 5.716, p = 0.023, η^2^= 0.152), especially within the visual cortex (F(20,640) = 5.207, p < 0.001, η^2^= 0.140). The fit increases toward the frontal cortex (F(20,640) = 6.114, p < 0.001, η^2^= 0.160).

Assessing the fit between the individual dissimilarity patterns within the different ROIs and the two representation models, the parametric increase in dissimilarity (1-r) with increase in distance between the match conditions in a representational space become apparent. Within early (F(1,32) = 18.085, p < 0.001, η^2^= 0.361) and extrastriate visual cortex (F(1,32) = 18.802, p < 0.001, η^2^= 0.370), this linearity within the model is larger than the estimate for the non-parametric deviant full-match representation. The only area with a larger estimate for the non-parametric regressor is the sensorimotor cortex (F(1,32) = 12.325, p = 0.001, η^2^= 0.278). Other comparisons within the remaining frontal and parietal areas do not yield significant main or interaction effects. The RDMs in the lower part ofFigure 5show a better discrimination between the full match and the full mismatch over time within parietal and frontal areas, causing a slight shift toward a better parametric fit towards T2. This is concordant with the SVM-results showing an increase in pairwise classification accuracy between M0 vs. M4 over time.

**Fig. 6.**
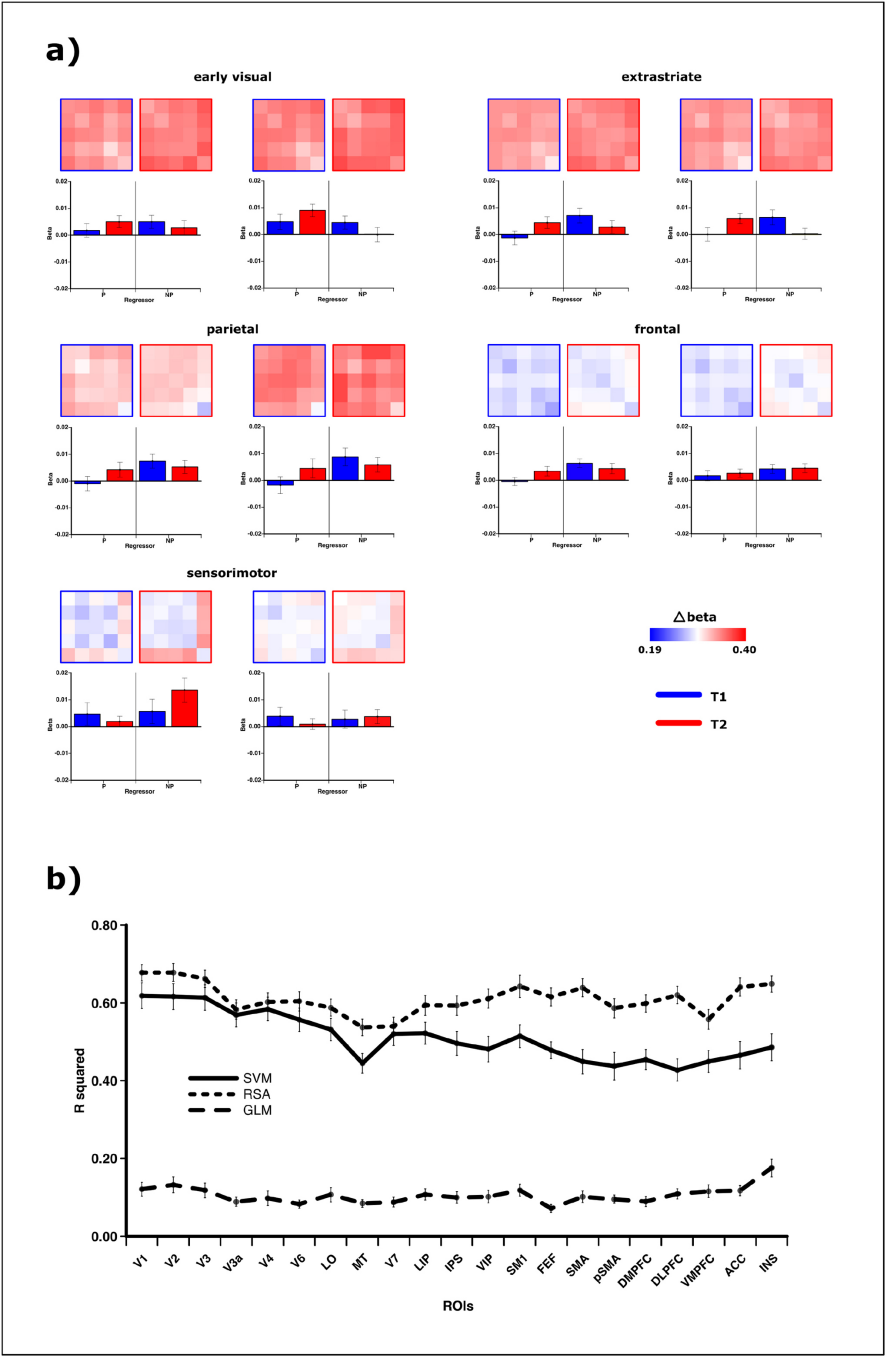
Univariate analysis. (a) The representational dissimilarity between the pairwise match-condition underlies their mean activation difference for the present univariate analysis. In summary, the parametric and non-parametric nature of the abstract representational space of the match condition is not well captured by the univariate analysis. (b) The univariate analysis does explain only around 10% of the variation in mean BOLD activation between conditions, whereas the multivariate approaches together with the parametric and non-parametric modeling can explain around 55–65% of the current data.

In order to estimate the performance of a univariate analysis in discriminating between the different match conditions, a second RSA was performed with the mean beta values across each examined ROI as a distance measure. This approach is equivalent to a GLM while directly comparable with the previous analyses. The performance for the regression models was quantified as overall explained variance (R^2^) and differed substantially (F(2,64) = 488.660, p < 0.001, η^2^= 0.939) between the analysis approaches (Fig. 6b). The combined parametric and non-parametric model explained hereby only around 10% of the variation within the dissimilarity patterns obtained by the mere mean activation of the GLM analysis. The SVM- and RSA-models, on the other hand, explained around 55% and 65%, respectively.

For the sake of completeness, the regression fits for the GLM were compared as well but did not yield any significant main or interaction effects across regressor, time, or hemisphere, except within the somatosensory ROI, which showed a better fit within the left hemisphere (F(1,32) = 6.381, p = 0.017, η^2^= 0.166), caused by a higher non-parametric regressor at T2 within the left hemisphere (t(32) = 2.160, p = 0.038, η^2^= 0.127). The dissimilarity matrices as well show that mean activation is not a good measure to capture the representational distances between the current target-probe conditions (Fig. 6a).

Finally, we sought to examine whether the observed functional patterns as well as changes within those patterns are related to subjects’ behavior and behavioral improvement and thus are specific to the administered training. For this purpose, we calculated a combination performance score to take both behavioral indicators of reaction time and error rates into account. We used the balanced integration score (BIS), which is a subtraction of the normalized accuracies and reaction times.

Location-based and object-based functional patterns, operationalized as the parametrical and non-parametrical model fits for the SVM analysis, were correlated with the BIS score for each session. Furthermore, the difference between the regressors over time was correlated with the change in the BIS score.

**Fig. 7.**
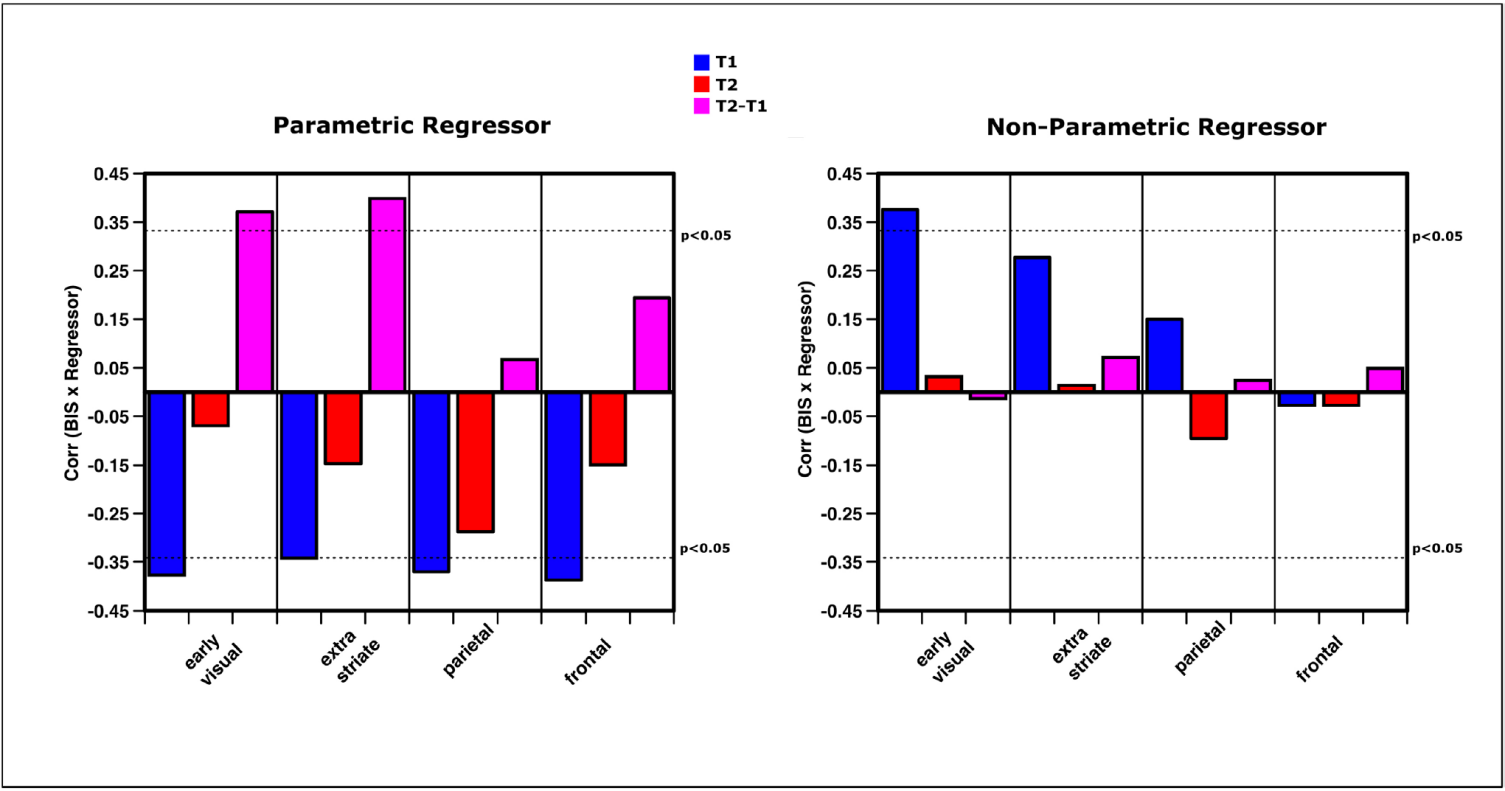
Correlations between functional model parameters and behavior. left – larger parametric regressors of the SVM analysis are associated with better performance throughout the cortex at the first scanning session. This correlation subsides toward the second scanning session. The behavioral training effect, on the other hand, is inversely related with the change in parametric fit. right—a reduced non-parametric fit within the visual cortex is associated with a better performance in the first scanning session. The dotted lines indicate the significance threshold at p < 0.05.

A higher initial tracking performance across subjects was related to an enhanced parametric pattern throughout the cortex (Early visual: r = -0.377, p = 0.031; extrastriate: r = -0.341, p = 0.052; parietal: r = -0.369, p = 0.034; frontal: r = -0.387, p = 0.026) (Fig. 7). Please note that a lower BIS points toward better performance. The non-parametric pattern was correlated with a lower general performance at T1 within early visual areas (r = 0.376, p = 0.031). Interestingly, activation patterns within location- and object-based functional networks were no longer correlated with behavior in the second scanning sessions. However, extenuated enhancements of the parametric fit within the visual cortex (Early visual: r =0.371, p = 0.033; extrastriate: r = 0.398, p = 0.022) were associated with a behavioral improvement from the first to the second session.

This may indicate a ceiling effect, in which the amount of adjustment of the parametric representation of the target determines the behavioral improvement, with subjects exhibiting optimized location-based representations profiting most of the training regimen.

## 4 Discussion

Tracking multiple visual objects over time is associated with neural activity in partly overlapping functional architectures devoted to the processing of location- and object-based attentional information across widespread cortical networks (Merkel et al., 2015). The current dataset investigates potential training-induced changes across these networks by comparing the difference in activity patterns before and after practice of the object-tracking task.

When performing the tracking task for the first-time, location-based information about the single relevant items is indexed by a parametric variation of neural activation patterns in higher-dimensional representational space differentially representing the target-probe congruities at the time of probe presentation. This parametric variation is strongly represented within the early visual and extrastriate cortices with a heavy bias toward the right hemisphere. At the same time the strength of this location-based representation is related to a better general performance.

Three days of training enhanced this parametric, location-based representation of the relevant items across visual, parietal, and frontal areas. However, the behavioral improvement over the same time-frame was associated with less modulation within these functional networks. On the other hand, highly distinct functional patterns associated with the representation of the configuration of the entire set of tracked objects were observed primarily within a widespread frontoparietal network with a left-hemispheric bias. In contrast to the location-based network, within the frontal functional networks training elicited a marked disengagement of the object-based representation.

### 4.1 Behavioral inter- and intra-subject variability in multiple object-tracking

Early discussions of determinants of tracking performance generally stated that subjects are able to track up to four items with an accuracy of around 85% (Cavanagh & Alvarez, 2005;Pylyshyn, 1989). On close inspection however, performance varies widely between subjects depending on individual resource availability. Early object-tracking work of Oksama and Hyönä, who consistently enrolled aviation-recruits for their studies, suggests that between-subject variability is potentially large in multiple object-tracking. Besides an on-average higher tracking capability, expert-participants exhibited a wider range of estimated individual capacity limits (2–6 items) (Oksama & Hyönä, 2004,^2008^) compared to the standard student population. This large variability in performing in the object-tracking task seems to be a result of specific long-term visuo-spatial perceptual experiences granting advantages in the object tracking task, which can be observed in other experts, namely flight-traffic control operators (Allen et al., 2004), 3D-video game players (Green & Bavelier, 2006), and some groups of experienced team-sport players (Jin et al., 2020;X. Zhang et al., 2009).

These findings suggest that performance in an object-tracking task potentially increases with long-term practice (Faubert & Sidebottom, 2012;Legault et al., 2013), although apparently with some limited transferability to other tasks. In a previous study, even novices exhibited a significant performance increase in object tracking after just three training sessions (Faubert, 2013), which is comparable to the training in the current dataset. Earlier studies in our lab suggested that performances toward different conditions in the present task-design are related to location (M0/M1/M2/M3)- and object(M4)-specific processes (Merkel et al., 2014,^2015^). The behavioral training effect of the present study seems to be unspecific in regards to those behavioral location- and object-based attentional processes, since subjects’ performances increase across all conditions with training.

### 4.2 Training effects on functional networks in multiple object tracking

The present functional imaging results align with a domain-unspecific behavioral training effect, as we observed changes in both functional networks exhibiting location- and object-related discrimination patterns for the probe display after prolonged training. Across subjects, a medial frontal disengagement for the network preferentially processing the probe-set as an object-based representation emerged. A marked decrease in overall BOLD-activation within prefrontal areas over the course of a 20 minutes object-tracking task was observed before (Tomasi et al., 2004). Similar frontal deactivations were found in repeated trials of a visual working memory task (Garavan et al., 2000). Additionally, successful object-tracking may be linked to a reduced functional connectivity within the dorsal attention network (Alnæs, Sneve, et al., 2015). Importantly, the disengagement of frontal networks was linked to improved performances in object-tracking tasks (Tomasi et al., 2014). While the current data are showing a diminished fit for the non-parametric representational model after training, this attenuation is, however, poorly related to a performance modulation. Object-based dissociation of the different match probes seemed to attenuate as the domain-specific task-set (identify full-matches) maintained within frontal areas like DLPFC, FEF, ACC, and SMA (Braver et al., 2003;Miller & Cohen, 2001;Pischedda et al., 2017) became less relevant and the responses toward the probe became more automated (Erickson et al., 2006;Landau et al., 2004) or underlay less attentional control (Corbetta & Shulman, 2002).

Within the occipital cortex, we observed an initially enhanced location-based discrimination pattern (parametric pattern) for subjects who performed well in the tracking task. This finding aligns well with a previously described positive relation between electrophysiological markers of attentional enhancement within the visual cortex and tracking performance (Stormer et al., 2013). The strengthening of the parametric functional pattern over time, however, was inversely related to the improvement elicited by training. While a generally larger engagement of the visual cortex in tracking tasks may be beneficial, an enhanced performance within the subjects may primarily rely on an optimization strategy causing reduced activity within relevant functional patterns of the visual cortex.

### 4.3 Functional mechanisms of visual perceptual learning

The functional enhancements within the visual sensory area most likely indicate perceptual learning effects elicited by a prolonged execution of the task (Karni & Sagi, 1991;Watanabe et al., 2002). Hereby, the locus of neural flexibility eliciting performance increases is thought to be determined by the relevant stimulus feature (Adab & Vogels, 2011;Ahissar & Hochstein, 2004;Schoups et al., 2001). Neuroimaging studies provide partly conflicting results regarding the nature of the neural changes in functional responses within the visual cortex associated with perceptual learning. Especially within the early visual system, BOLD-increases in the context of perceptual learning (Furmanski et al., 2004;Maertens & Pollmann, 2005;Schwartz et al., 2002), as well as BOLD-decreases (Mukai et al., 2007;Schiltz et al., 1999) have been described in similar experimental setups. However, classification approaches seem to be able to resolve this contradiction by showing that learning effects within the visual system appear as sharpened tuning toward relevant stimulus categories (Desimone, 1996;Schoups et al., 2001). Therefore, perceptual learning may result in a reduced overall BOLD-signal within domain-specific areas processing the task-relevant stimuli, which at the same time allows for an increased discriminability within the relevant domain (Jehee et al., 2012;Roth & Zohary, 2015). The present data demonstrate this dissociation between univariate and multivariate approaches by showing that differences between match-conditions are embedded within a higher-order representational space, while the overall BOLD activation level remains constant and a poor predictor of the differences between conditions. For the current classification-approach, early visual changes for the parametric model may reflect modulations in the location-specific encoding of the single target-items after training. One may speculate that the observed negative relation between location-based representational enhancement and reduced behavioral training effects may indicate a shift of pertinence toward the abstract object-representation, which may help to obtain the required full- vs. no-full-match decision. In the same vein, the modification of visual experience by perceptual learning does involve the optimization of representations by extracting task-relevant image regularities (Kourtzi, 2010;Schwarzkopf & Kourtzi, 2008;Zhang & Kourtzi, 2010).

### 4.4 Two functional networks in multiple object tracking

In general, widespread functional networks across the cortex responsible for control and performance in the tracking task involve frontal, parietal, and extrastriate areas (Alnæs, Kaufmann, et al., 2015;Culham et al., 1998;Howe et al., 2009;Mäki-Marttunen et al., 2019). The concept of two distinct functional neural networks related to the general attentional engagement during tracking on the one hand and the attentional load defined by the number of relevant targets on the other hand is widely accepted (Culham et al., 2001;Jovicich et al., 2001). In this view, a pure tracking-related frontoparietal network exhibits a modulation in BOLD responses during active tracking of multiple objects independent of attentional load and was described to largely overlap with the dorsal attention network (Corbetta et al., 2008) involving SPL, MT, FEF, and DLPFC (Culham et al., 1998,^2001^;Howe et al., 2009). The involvement of this network, usually controlling endogenous attentional shifts (Corbetta, 1998;Serences & Yantis, 2007), may establish a continuous engagement in the task throughout active tracking (Alnæs, Kaufmann, et al., 2015) and steady eye-movements toward the centroid of the target-set (Fehd, 2010;Howe et al., 2009;Nummenmaa et al., 2017) or the fixation (Burman & Bruce, 1997). The second network aligns with the general idea of indexing in multiple-object-tracking, in that individual spatial pointers to the locations of the relevant objects need to be maintained during tracking (Alvarez & Franconeri, 2007;Pylyshyn, 1989). Accordingly, this system, centered around IPS and right prefrontal cortex (Culham et al., 2001) including right SMA (Culham et al., 2001;Mäki-Marttunen et al., 2019) and the anterior cingulate (Jovicich et al., 2001), exhibits a parametric variation of BOLD enhancement depending on the amount of relevant information to be tracked (load-dependent activation).

Our current results as well as earlier observations (Merkel et al., 2015) partly align with this general notion of such a load-dependent and load-independent tracking system. Importantly, the current classification-approach reveals contributions within different functional networks with respect to the discriminability between different conditions rather than general BOLD increases or decreases. Furthermore, the different conditions throughout the experiment are neither different in their ‘attentional load’ nor in the ‘mental effort’ during tracking (subjects have to track always four out of eight items). The difference consists in the visual match between a four-item probe with the tracked four-item target set. Therefore, regions exhibiting a parametric representation (IPS, striate- and extrastriate areas) would partly serve the function of a classical load-dependent area insofar as a multi-item probe is matched*individually*against the attended four-item target, based on location-congruity. And although the occipital cortex typically does not show load-dependent BOLD modulations during the tracking task (Culham et al., 2001;Jovicich et al., 2001;Mäki-Marttunen et al., 2019), the current data suggest that retinotopic areas of the striate and extrastriate cortex encode information about the spatial overlap between a set of continuously attended locations and a following sensory input (probe). Accurate classification in the absence of univariate BOLD-modulations for distinct stimulus material is not very surprising, since it was observed in working memory tasks before (Linden et al., 2012;Serences et al., 2009). Interestingly, this observed occipital network exhibits a strong right-hemispheric bias, aligning with the notion of a location-based tracking system implemented predominantly within the right hemisphere (Lesch et al., 2020;Merkel et al., 2024).

Areas exhibiting activity with a significant fit for the non-parametric model on the other hand process object-based information of the entire target set. The engagement of this network is entirely load-independent as its all-or-nothing response-pattern would only encode object-based information related to probes that fully overlap with the target set. The dorsal attention network related to the general tracking task described earlier (Culham et al., 2001;Howe et al., 2009) incorporates parts of the non-parametric representation network observed here. This is highly consistent with our interpretation, as object-based attentional control engages the very same frontal (ACC, DLPFC, FEF, SMA) and parietal (IPS, SPL) regions (Arrington et al., 2000;Shomstein & Behrmann, 2006;Wilson et al., 2005). The medial frontal part of the frontal cortex was previously associated with attentional capture of objects over locations (Stoppel et al., 2013). In general, the current cortical distribution of distinct BOLD-patterns (object- and location-based) recorded during the first MR-session confirms previous results (Merkel et al., 2015) and may be functionally aligned with the distinction of a load-dependent and load-independent tracking network.

## 5 Conclusion

In conclusion, repeated training of the multiple-object-tracking task elicited complex modulations within two partly overlapping neural networks over time, highlighting task-specific neural flexibility. The neural underpinnings of the improvement of visual tracking performance assessed with a probe at the end of the tracking period involve differential consolidations of location-based representations within the visual system as well as a disengagement of frontal control structures for object-based representations.

## Data Availability

All data on which the results and conclusions are based on as well as the custom scripts used are available on the repository:https://osf.io/v67ya
